# Osteological, multi-isotope and proteomic analysis of poorly-preserved human remains from a Dutch East India Company burial ground in South Africa

**DOI:** 10.1038/s41598-023-41503-9

**Published:** 2023-09-06

**Authors:** Judyta Olszewski, Rachael A. Hall, Lisette M. Kootker, Neil J. Oldham, Robert Layfield, Barry Shaw, Leon Derksen, Martijn Manders, Tim Hart, Sarah A. Schrader

**Affiliations:** 1 Laboratory of Human Osteoarchaeology, Faculty of Archaeology, Leiden University, Leiden, The Netherlands; 2 Centre for Applied English Studies, Faculty of Arts, The University of Hong Kong, Pokfulam, Hong Kong SAR, Hong Kong; 3 Department of Earth Sciences, Geology and Geochemistry Cluster, Faculty of Science, Vrije Universiteit Amsterdam, Amsterdam, The Netherlands; 4 CLUE+ Research Institute for Culture, History and Heritage, Vrije Universiteit Amsterdam, Amsterdam, The Netherlands; 5 School of Chemistry, University of Nottingham, University Park, Nottingham, NG7 2RD UK; 6 School of Life Sciences, University of Nottingham, Nottingham, UK; 7 ACO Associates, Cape Town, South Africa; 8 Cultural Heritage Agency of the Netherlands, International Maritime Heritage, Amersfoort, The Netherlands

**Keywords:** Archaeology, Biological anthropology, Proteomics, Geochemistry, Biogeochemistry

## Abstract

Skeletal remains discovered in Simon’s Town, South Africa, were hypothesised as being associated with a former Dutch East India Company (VOC) hospital. We report a novel combined osteological and biochemical approach to these poorly-preserved remains. A combined strontium (^87^Sr/^86^Sr), oxygen (*δ*^18^O_VPDB_) and carbon (*δ*^13^C_VPDB_) isotope analysis informed possible childhood origins and diet, while sex-specific amelogenin enamel peptides revealed biological sex. Osteological analyses presented evidence of residual rickets, a healed trauma, dental pathological conditions, and pipe notches. The combined isotope analyses yielded results for 43 individuals which suggested a diverse range of geological origins, including at least 16% of the population being non-local. The inclusion of *δ*^13^C_VPDB_ had intriguing implications for three individuals who likely did not have origins in the Cape Town region nor in Europe. Peptide analysis on the dental enamel of 25 tested individuals confirmed they were all biologically male. We suggest that isolated enamel may provide crucial information about individuals’ pathological conditions, geographical origins, diet, and biological sex. These data further demonstrated that a combined approach using multiple osteological and biochemical methods is advantageous for human remains which are poorly preserved and can contextualise a site with little direct evidence.

## Introduction

**Figure 1:**
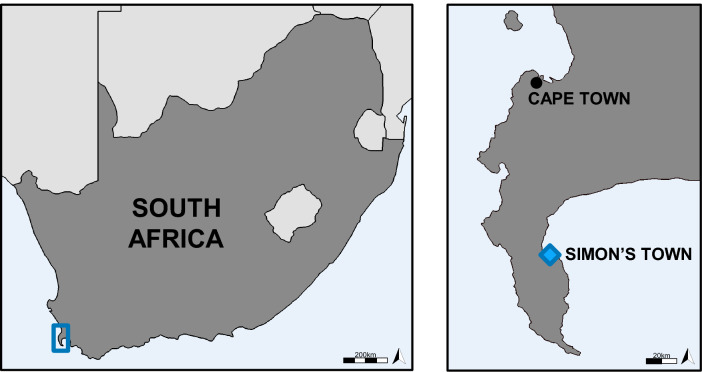
Map of South Africa, and map of Cape peninsula region with Simon’s Town highlighted. Maps compiled and created by authors.

Poor preservation of archaeological remains due to temporal and taphonomic factors introduces challenges for many researchers^1^. In the context of human remains, many macroscopic osteological techniques are only informative if the skeletal elements are complete enough to allow for accurate measurements. As a result, the introduction of biochemical techniques, such as isotope and proteomics analysis, has expanded possibilities to obtain information from poorly-preserved remains on an individual and population level, and are being conducted more frequently in recent research^2,3^. In Simon’s Town, South Africa (Fig. 1), the poorly-preserved remains of 184 individuals were excavated from an inactive and unmarked burial ground during routine archaeological assessments, raising questions about their history and context. The structural integrity of the bone excavated from the site was consistently poor, to such a degree that it would crumble upon excavation, including during en bloc excavation attempts. The poor preservation prevented the application of conventional osteological analytical techniques (i.e., estimating osteological sex, age-at-death, stature, and identification of pathological conditions). Archival research into the excavation site unearthed evidence of an inactive burial ground associated with a previously standing Dutch East India Company (VOC) hospital in use between 1765 and 1795 CE. The hospital largely catered to sick VOC personnel from ships calling at Simon’s Bay, now False Bay, during the winter months when weather conditions at Cape Town made it too dangerous to anchor. Occasionally, the hospital admitted personnel from ships of other trading and naval companies^4^. Globally, few VOC specific cemeteries have been identified and excavated^5,6^. Therefore, the motivation behind this research was to identify whether the Simon’s Town site was indeed a VOC hospital burial ground principally servicing VOC personnel, potentially elucidating valuable insights into Dutch and South African historic relations and further contributing to global proteomic and isotope databases. This was examined through a combined osteological, archaeological, archival, multi-isotopic, and proteomics approach.

If the burial ground was a part of the VOC hospital, the expectation would be to find predominantly male adults as the VOC exclusively hired males, and children under 14 years old were rarely employed^7–9^. Conversely, if the burials represented a local population the expectation would be a mixed distribution of sexes and ages, and there would be a notable presence of non-adults due to the high infant mortality rates at the time^10–12^. The demographics would include settler communities, globally employed VOC sailors and soldiers, as well as local indigenous communities or communities of enslaved peoples from throughout the Dutch and later British occupations, as observed in other excavated contemporary sites in the region^7,13,14^. By the mid-1700s, the Dutch colony was already well-established in the Cape^15^. Consequentially, what is considered 'local' during this time is complex, especially with regards to skeletal isotope chemistry. Those indigenous to the Cape, and those born and raised in the Cape as second-generation colonisers or enslaved peoples would register the same isotopic data. Moreover, those who were displaced into the Cape after childhood, both voluntarily and involuntarily, would create a population with more diverse origins than a fully local population. Therefore, comparing demography with geographical origins to understand more about the population is highly meaningful, given the potential VOC context and the complex history of colonisation in the region^16–18^.

Addressing questions of the individuals’ origins contributed to overarching investigations of the site by utilising a combined funerary archaeological, osteological, proteomics, and isotopic approach. Osteological examination and the increasingly popular technique of proteomics to identify sex-specific tooth enamel proteins provide context into population demographics for the buried individuals. Funerary archaeology focusing on burial styles and associated artefacts provide further context to potential differing local *versus* non-local socio-religious contexts. Combined strontium (^87^Sr/^86^Sr), oxygen (*δ*^18^O_VPDB_), and carbon (*δ*^13^C_VPDB_) isotope analyses using tooth enamel informs about childhood geographical origins and dietary preferences by comparing the data to expected local values from the Cape Town region of South Africa^13,19–21^. As isotope analyses can only confirm non-local origins through deviations of expected ranges, any individuals which fall within expected ranges are assumed local, despite the possibility of alternative interpretations given the wide range of strontium ratios for the Cape region.

In this paper, the results of the osteoarchaeological, funerary, multi-isotope, and proteomics research are presented. These data uniquely contribute to a larger research project that investigates the context of the Simon’s Town site by providing empirical data to support the limited historical sources in ongoing research. Further, this research contributes to the low recovery rate of VOC-related burial grounds, while illustrating the importance and future potential of biochemical techniques when used on poorly-preserved skeletal remains. Finally, we demonstrate the first known large scale multimethod approach in bioarchaeological literature that combines data from historical archives, archaeological contexts, macroscopic osteology, multi-isotope, and peptide analysis to interpret both the individual and population level context for poorly-preserved skeletal remains.

## Results

All information from the funerary, osteological, and biomolecular analyses of the 43 individuals analysed for this research is presented in Table 1.

**Table 1:** Osteological, proteomic, archaeological, and isotopic data of first and second molars, collected from 43 analysed individuals excavated from the Simon’s Town, South Africa burial site.

Burial #	Tooth*^1^	Sex*^2^	Age category	^87^Sr/^86^Sr	2SE	δ^13^C/‰ (apatite, VPDB)	s	δ^18^O/‰ (apatite, VPDB)	s	δ^18^O/‰*^3^ (VSMOW)	Associated artefacts
6	27	–	Adult	0.709279	0.000008	− 10.6	0.1	− 6.4	0.1	24.3	–
9	36	M	Adult	0.710685	0.000006	− 9.8	0.1	− 5.8	0.1	25.0	–
10A	16	M	Adult	0.709348	0.000010	− 9.9	0.1	− 5.4	0.2	25.3	–
15	36	–	Adult	0.711473	0.000008	− 11.2	0.1	− 5.2	0.1	25.6	–
18B	36	M	Adult	0.712245	0.000007	− 13.6	0.1	− 4.2	0.1	26.5	–
18D	46	M	Adult	0.709654	0.000007	− 13.3	0.1	− 6.6	0.2	24.1	–
20	27	–	Adult	0.709252	0.000007	− 10.5	0.1	− 8.0	0.2	22.6	–
22C	26	–	Adult	0.712724	0.000010	− 11.2	0.1	− 7.3	0.1	23.4	–
27	37	M	Adult	0.710015	0.000008	− 10.3	0.1	− 6.5	0.1	24.2	Leather, wooden coffin
33	26	M	Adult	0.710826	0.000008	− 10.8	0.1	− 7.2	0.3	23.5	–
36A	36	M	Adult	0.711804	0.000007	− 12.3	0.0	− 6.2	0.1	24.5	–
42B	26	–	Adult	0.707589	0.000010	− 1.9	0.0	− 3.6	0.1	27.2	–
43A	36	M	Adult	0.708839	0.000010	− 11.9	0.1	− 6.7	0.1	24.0	–
43B	37	M	Adult	0.711499	0.000007	− 13.7	0.1	− 4.9	0.1	25.9	–
43C	16	M	Adult	0.710061	0.000006	− 10.8	0.1	− 6.4	0.2	24.3	–
43D	36	M	Adult	0.707409	0.000006	− 9.4	0.1	− 5.9	0.1	24.9	–
48	16	M	Adult	0.710458	0.000009	− 10.6	0.1	− 10.0	0.2	20.6	–
49	36	M	Child	0.711553	0.000009	− 10.6	0.1	− 5.4	0.2	25.4	–
53	46	M	Adult	0.709828	0.000007	− 13.2	0.1	− 5.9	0.1	24.9	–
58	46	M	Adult	0.709519	0.000008	− 11.5	0.1	− 7.6	0.1	23.1	–
59	26	M	Adult	0.709791	0.000008	− 10.6	0.1	− 5.9	0.1	24.8	Buttons
60A	47	M	Adult	0.710057	0.000009	− 10.8	0.0	− 5.6	0.1	25.1	–
61	46	–	Adult	0.709846	0.000008	− 9.3	0.1	− 5.3	0.1	25.4	–
65	16	M	Adult	0.709796	0.000007	− 10.0	0.1	− 5.9	0.2	24.8	–
66	26	M	Adult	0.715812	0.000008	− 7.9	0.0	− 3.5	0.1	27.4	–
67	37	M	Adult	0.709221	0.000008	− 10.3	0.1	− 5.9	0.1	24.8	–
69	36	M	Adult	0.709607	0.000008	− 13.4	0.0	− 5.8	0.1	24.9	–
72	16	M	Adult	0.710528	0.000009	− 3.8	0.1	− 6.6	0.2	24.1	Buttons, wooden coffin
75	26	–	Adult	0.709393	0.000008	− 10.1	0.1	− 6.0	0.2	24.7	–
95	46	–	Adult	0.709522	0.000009	− 9.7	0.1	− 7.1	0.2	23.6	–
97A	16	–	Adult	0.711981	0.000006	− 11.9	0.0	− 7.7	0.1	23.0	Button with anchor motif (possibly from 97B)
97B	36	M	Adult	0.709785	0.000019	− 13.9	0.0	− 6.8	0.1	23.9	Bead necklace (36 green/white striped, 5 black)
98	26	–	Adult	0.711448	0.000008	− 10.8	0.1	− 7.6	0.2	23.1	–
100	16	–	Adult	0.709300	0.000006	− 10.1	0.1	− 6.8	0.2	23.9	–
105	46	–	Adult	0.709102	0.000008	− 5.3	0.1	− 6.5	0.1	24.3	–
107	46	M	Adult	0.709358	0.000006	− 13.2	0.1	− 7.2	0.1	24.9	Buttons, scissors
108	16	–	Adult	0.709957	0.000008	− 12.1	0.1	− 5.1	0.1	25.7	Bone buttons
113	46	–	Adult	0.709282	0.000013	− 9.6	0.1	− 6.9	0.2	23.9	–
115A	16	–	Adult	0.710019	0.000008	− 9.5	0.1	− 8.4	0.1	22.2	–
118	17	–	Adult	0.709363	0.000008	− 10.7	0.1	− 6.7	0.2	24.0	–
121	16	–	Adult	0.711503	0.000009	− 12.2	0.1	− 6.7	0.1	24.0	Buttons (11 total)
122A	26	–	Adolescent	0.709254	0.000008	− 10.4	0.1	− 7.5	0.1	23.2	Button hook
138	16	M	Adult	0.712174	0.000008	− 1.3	0.1	− 5.0	0.2	25.8	–

*2SE* 2 standard error, *s* standard deviation.

*^1^All teeth are identified by the FDI World Dental Federation notation (ISO 3950).

*^2^Chromosomal sex from peptide analysis results of this research.

*^3^VSMOW values were obtained from conversion equations as outlined in Coplen 1998.

### Funerary practices

The predominant burial style for the 184 individuals was supine, with the arms positioned either along the sides of the body or crossed over the pelvis or torso (*123*/184) (Table 2). Few individuals deviated from this burial style (*10*/184), but there remain unknown burial styles due to partially or fully disarticulated burials (*51*/184). There was also considerable evidence of grouped (mass) burials (*n* = 20) containing two or more individuals (*n* = 51 individuals in total). Additionally, disorganised placement of graves resulting in frequent secondary deposition (i.e., displaced and disassociated bones atop or near complete burials). Seven coffin burials were identified through remnants of preserved wood, coffin silhouettes, and coffin nails. A few burials had associated artefacts (*27*/184), which included buttons, button hooks, pins, small textile fragments (leather, cloth, rope), a glass trade bead necklace (black, green, and white striped), as well as numerous fragmentary pipe stems and pipe bowls, of which one had a maker’s mark from Gouda, The Netherlands. Two European coins were associated with burial 37C, one of which was identifiable as a seventeenth-century VOC 6 *stuiver*. An eighteenth-century French silver *écu* was associated with burial 74.

**Table 2:** Burial styles of the 184 excavated individuals from Simon’s Town, South Africa burial site.

Burial style	No. individuals
Supine, arms by side	10
Supine, arms crossed over pelvis	43
Supine, arms crossed over abdomen/thorax	56
Supine, arms alternate over pelvis/abdomen	14
Other (e.g. extended but on side, prone)	10
Disarticulated individuals	20
Unobservable	31
**Total**	**184**

Coffin vs. no-coffin	
Coffin burials	8
Non-coffin burials	156
Other (i.e. disturbed, disarticulated)	20
**Total**	**184**

### Age assessment

Osteological age-at-death estimations were limited to broad group classifications of non-adults (≤ 20 years) or adults (> 20 years)^22^, due to poor preservation. Gross osteological observations estimated that 178 individuals were adults, and six individuals were identified as non-adults using dental eruption, dental formation, and epiphyseal fusion^22–25^ (Table 3). A mandible fragment from one of the non-adults was found in a disarticulated bone pile and was the only apparent fragment representing this individual. The majority (*5*/6) of non-adults ranged from 11 to 20 years of age, while the youngest individual was approximately 3 to 6 years old.

**Table 3:** Age distribution of 184 excavated individuals from Simon’s Town, South Africa burial site.

Age category*	Age range (in years)	No. individuals
Adult	> 20	178
Adolescent	12–20	4
Child	3–12	2
Infant	0–3	0
**Total**		**184**

*Age categories defined according to Buikstra and Ubelaker^22^.

### Pathological conditions

In most cases, macroscopic assessment of the skeletal remains was not possible. The only identifiable pathological conditions were residual rickets^26^ and evidence of a healed ankle fracture. Dental assessments for 22 individuals consistently revealed typical dental pathologies (*21*/22). These affected individuals exhibited evidence of caries (*n* = 18 individuals), calculus (*n* = 15 individuals), enamel hypoplasia (*n* = 13 individuals), and antemortem tooth loss (*n* = 6 individuals). Although not pathological, pipe notches were also observed in four individuals' maxillary and mandibular anterior and premolar teeth. There were no observations of intentional dental modifications in the form of decorative filing or tooth evulsion as prevalent amongst numerous African^27,28^ and Indian Ocean basin^29^ populations.

### Proteomics

**Figure 2:**
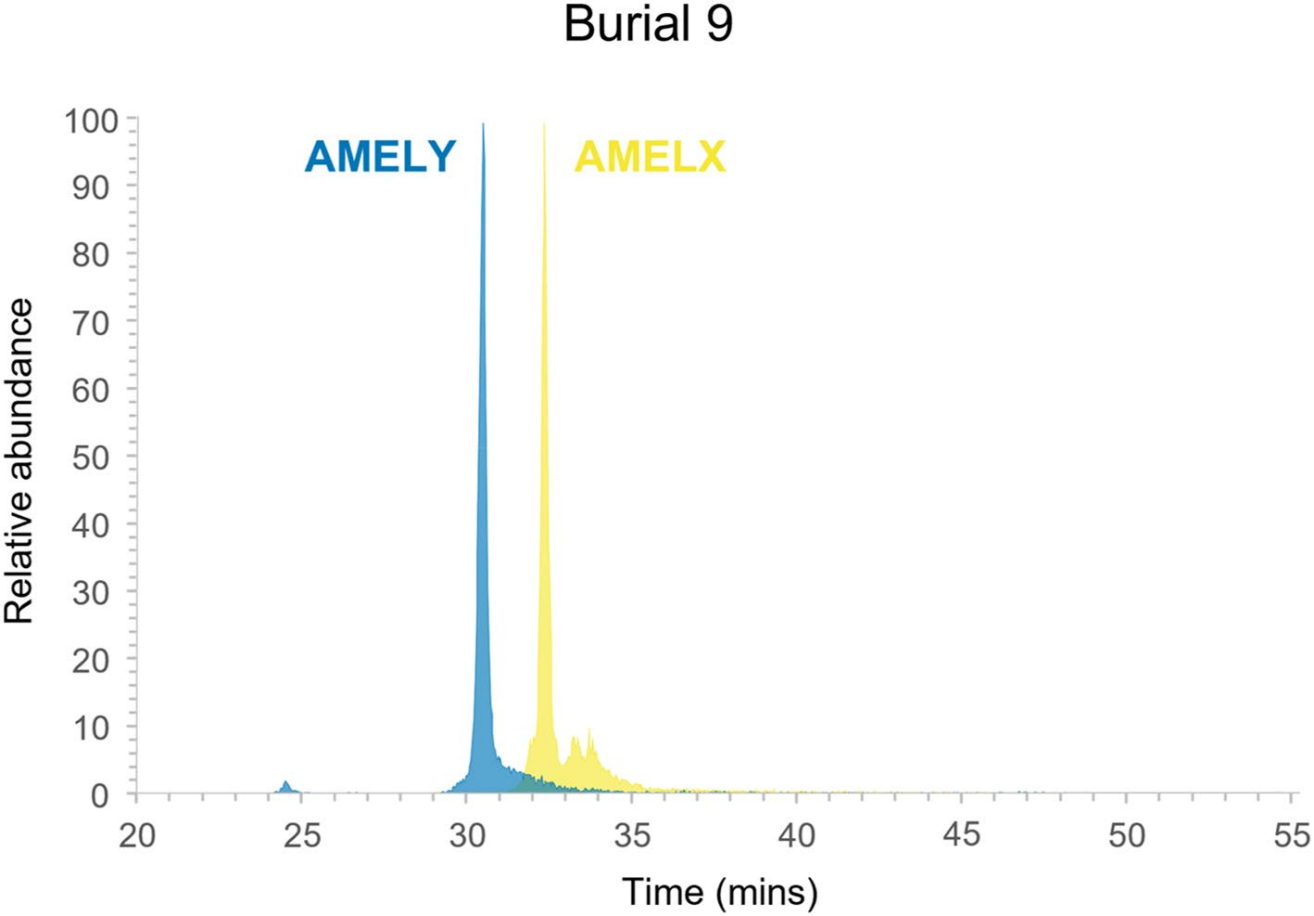
Reconstructed ion chromatograms demonstrating detection of both AMELX and AMELY peptides from enamel extract of tooth from burial 9. Data are representative of results for all 25 samples analysed.

Mass-spectrometry based peptide analysis was performed on the tooth enamel of 25 randomly selected individuals to identify sex chromosome-linked protein isoforms. Peptides specific to the X-chromosome amelogenin isoform (AMELX peptide sequence = SIRPPYPSY) and the Y-chromosome isoform (AMELY peptide sequence = SM(ox)IRPPY) were consistently detected in all individuals. Thus, in this research with poorly-preserved remains, the detection of both AMELX and AMELY peptide sequences strongly demonstrated that all 25 sampled individuals were chromosomally male (Fig. 2).

### Strontium, oxygen, and carbon isotopes

**Figure 3:**
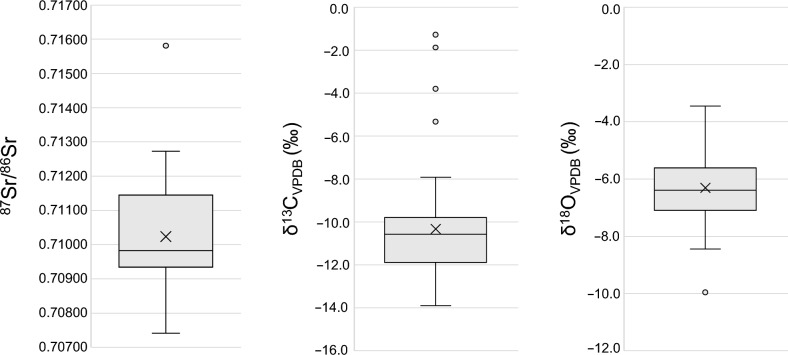
Boxplots of isotope data for 43 analysed individuals from Simon’s Town, South Africa burial site. Key: Whiskers represent minimum and maximum values of data according to the interquartile range rule. The top box represents the 3rd quartile, and the lower box represents the 1st quartile. The top box line represents the median of the 3rd quartile, the middle line represents the median of the whole dataset, and the lower box line represents the median of the 1st quartile. The × visible within the box represents the mean. All data points that are present outside of the whisker range represent suspected outliers.

Table 1 presents ^87^Sr/^86^Sr, *δ*^18^O_VPDB_ and *δ*^13^C_VPDB_ data obtained from 43 randomly selected individuals. Boxplots present and visualise the spread of the data and identify outliers (Fig. 3).

**Figure 4:**
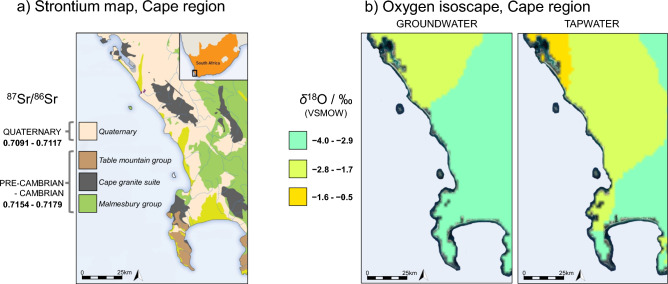
Expected local (**a**) strontium ratios*^1^ and (**b**) oxygen values*^2^ from water sources in the Cape region of South Africa, as referenced in this research to develop expected local ranges. *^1^Image (**a**) Schematic geological map representing archaeological fauna and human Sr values from Sealy et al.^21^, Balasse et al.^30^, and Radloff et al.^31^. Image modified from Kootker et al.^34^; Fig. 2. Schematic geological map of the southwestern Cape. *^2^Image (**b**) Modified image from West et al.^20^ isoscape, developed with geospatial and oxygen data.

Published strontium isotope ratios from relevant archaeological faunal and human data were assessed to identify baseline strontium ranges for the region. The entire Cape Town region is geologically represented by Quaternary sand, Precambrian Malmesbury Precambrian Cape granite suite, and Cambrian Table Mountain groups (Fig. 4). Published bioavailable ^87^Sr/^86^Sr data from both herbivorous non-migratory faunal enamel, and directly comparable human enamel was used to determine local ^87^Sr/^86^Sr ranges. The entire Cape Town region has a wide ^87^Sr/^86^Sr range (0.7091–0.7179)^21,30–33^. The analysed individuals for this research produced a range of 0.7074 to 0.7158. Consequently, most analysed individuals (*39*/43) exhibited ratios congruent with local Cape Town ranges. In contrast, the ^87^Sr/^86^Sr of three individuals (burials 42B, 43A, and 43D) were lower than the expected Cape Town range, ranging from 0.7074 to 0.7088, and were thus considered non-local.

Dietary expectations for the Cape Town region during the seventeenth to eighteenth centuries suggested a predominance of C_3_ plant consumption, with some inclusion of C_4_ plants. A *δ*^13^C range of analysed dentine from contemporary sites suggested a local dietary range of − 18.8‰ to − 13.5‰^34^, corresponding with a significantly higher contribution of C_3_ foods than C_4_^13^. Further, human enamel carbonate data from pre-contact and VOC contemporary Cape Town populations are consistent with that of *δ*^13^C from dentine, with a *δ*^13^C_VPDB_ range of − 13.4‰ to − 6.1‰, representing a C_3_ to C_3_–C_4_ mixed diet^13,19^. Notably, enamel and collagen isotope analyses in prior studies have used *δ*^13^C values suggestive of C_4_ diets as a proxy for non-local origins, alongside dental modifications and ^87^Sr/^86^Sr^13^.

The *δ*^13^C_VPDB_ data range from − 13.9‰ to − 1.3‰. The *δ*^13^C_VPDB_ of most individuals (*36*/43) fell within a range of − 12.3‰ to − 7.9‰, indicating a C_3_ dominant to mixed C_3_–C_4_ diet, which is consistent with comparable enamel data from the region^13,19^, Three burials (18B, 43B, and 97B) had low *δ*^13^C values, indicating C_3_ dominant diets just outside the established local range (− 13.9‰ to − 13.6‰). However, C_3_ values may be influenced by numerous factors including canopy type, water levels^35,36^. Therefore, burials 18B, 43B, and 97B were considered local, whereas four individuals (burials 42B, 72, 105 and 138) who consumed C_4_ dominant diets (− 5.3‰ to − 1.3‰), were suggested to be of non-local origins.

The *δ*^18^O values from tooth enamel represent the oxygen isotopic composition of ingested and imbibed water. While this includes plant and animal foods, and respiration, the highest proportional contribution to the *δ*^18^O values comes from imbibed water^37^. Therefore, precipitation in the Cape region was reviewed to assess the expected *δ*^18^O ranges for the Cape region using the Global Network of Isotopes in Precipitation (GNIP) database, combined with other published precipitation, ground and tap water *δ*^18^O values, to create an expected range of *δ*^18^O values for the region^38,39^ (Fig. 4). Precipitation data for the region produced a conservative *δ*^18^O_VSMOW_ range of − 4.7‰ to 0.75‰ for the Cape Town region^20,40–42^. The 43 individuals assessed in this research produced *δ*^18^O_VPDB_ values ranging from − 10.0 to − 3.5‰. Using *δ*^18^O has limitations, therefore, precautions were implemented by applying pretreatment protocols when utilising *δ*^18^O values in the identification of non-local values^43,44^. In addition, prioritising first molars to elucidate the earliest childhood origins may result in the *δ*^18^O data being offset by ^18^O-enrichment due to breastfeeding during tooth development^45,46^. Researchers have noted that human enamel *δ*^18^O values may vary up to 2‰ within a single individual^35^, and larger differences have been reported at an intrapopulation level (potentially > 3‰)^47^. Additionally, recent studies have suggested that extra care should be taken when using precipitation values as proxies for imbibed water, as large variations can be observed in *δ*^18^O_enamel-carbonate_ depending on the region and environment^39^. There is little known about the intra-dental elemental variation of *δ*^18^O values from this region and period, which may influence the interpretation of *δ*^18^O_VPDB_ values^35^. Consequently, *δ*^18^O values are presented but were evaluated carefully, and only values which were considerably lower than might be expected for the region have been suspected as non-local given regional climate and precipitation *δ*^18^O values.

**Figure 5:**
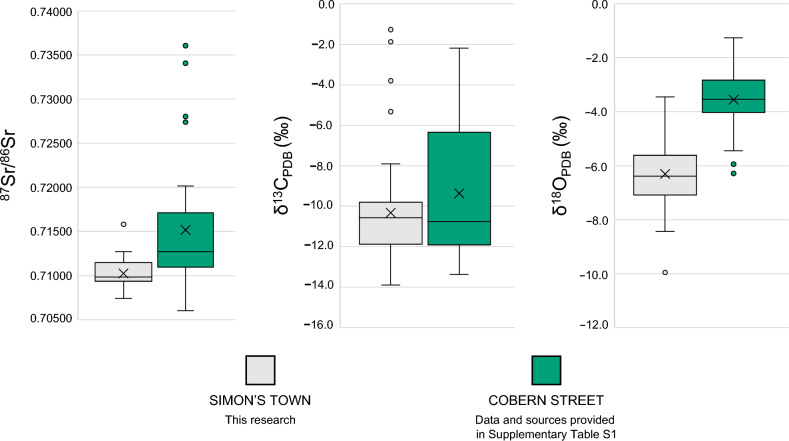
Box plots comparing isotope data between 36 individuals from the Cobern Street, South Africa burial site, and 43 individuals from the Simon’s Town, South Africa burial site. Key: Whiskers represent minimum and maximum values of data according to the interquartile range rule. The top box represents the 3rd quartile, and the lower box represents the 1st quartile. The top box line represents the median of the 3rd quartile, the middle line represents the median of the whole dataset, and the lower box line represents the median of the 1st quartile. The × visible within the box represents the mean. All data points that are present outside of the whisker range represent suspected outliers.

A direct comparison between *δ*^18^O_VPDB_ data from the enamel of a contemporary population was made to better understand expectations for local populations. Therefore, we compared our results to previously unpublished human enamel *δ*^18^O_VPDB_ data of 35 individuals from the contemporary colonial Cobern Street burial ground in Cape Town (Supplementary Table S1). This provided coeval demographic comparisons between isotope ranges and value distributions. The Cobern Street burial ground represents a population of locally born lower-class citizens and enslaved persons (locally born or forcibly displaced) from the eighteenth century^11,34,48^. Cobern Street individuals *δ*^18^O_VPDB_ values range from − 6.3 to − 1.9‰. There is a clear difference when comparing the spread of the data and the mean/median values (Fig. 5).

Results from an independent two-tailed *t*-test presented statistically significant differences between the oxygen ranges of Cobern Street (M = − 3.55, SD = 1.05), and this research (M = − 6.31, SD = 1.23); *t*(79) = 10.874, *p* = < 0.001. Such dissimilarity in the oxygen isotope ranges between sites can tentatively suggest the Simon’s Town population of this research came from significantly different climactic origins than those of the Cobern Street population. In particular, burial 48 with a* δ*^18^O_VPDB_ value of − 10.0‰, seems highly unlikely to be local given the coastal proximity, altitude, climate and precipitation levels in the Cape region. However, it is noted that small differences in the data could be the result of differing pretreatments, as the Cobern Street enamel was not pretreated^43,44^. Therefore, it must be stressed that if local human enamel *δ*^18^O_VPDB_ data are published, or the relationship between precipitation and *δ*^18^O_carbonate_ from the Cape region is investigated, this evaluation of Simon’s Town *δ*^18^O_VPDB_ values should be revised^39^.

While the geologically diverse Cape Town region resulted in a broad ^87^Sr/^86^Sr baseline range, three individuals were identified as non-local (burials 42B, 43A, and 43D; a range of 0.7074 to 0.7088). The addition of *δ*^18^O and *δ*^13^C isotopes aided in identifying further non-locals, which for *δ*^18^O included burial 48 (− 10.0‰), and for *δ*^13^C included burials 42B, 72, 105, and 138 with a C_4_ dominant diet (− 5.3‰ to − 1.3‰). These results suggest that at least 16% (*7*/43) of the analysed individuals were non-local to the Cape Town region. The statistically significant difference between the oxygen data from Cobern Street and this research may suggest an underestimation of the extent of non-local origins in this population.

## Discussion

Demographic assessments in the form of sex, age, and associated material culture revealed two distinct demographic aspects that suggested a relation to the VOC hospital. The 25 individuals analysed for sex-specific peptides all had isoforms derived from both X and Y chromosomes. Identifying the AMELY peptide, SM(ox)IRPPY, in all tested individuals strongly suggested that all assessed individuals were biologically male, supporting the hypothesis that the site likely represented VOC personnel. To further investigate the significance of the sex distribution, we compared our results to the demography of previously excavated contemporary burial sites from Cape Town. For example, the Cobern Street burial site reported a slight skew towards male presence over females (59% male, *n* = 63 adults)^48^. Additionally, a predominance of the male sex was observed at the Marina Residence hospital burial site (72% male, *n* = 72 adults)^10,11^. We stress, however, that the complete absence of identified females in this research is distinct. Further, while the majority were adults, six non-adult individuals were identified, with one young child (aged 3 to 6 years) and the remaining non-adults presenting ages of 11 to 20 years. While the employment of adolescents is consistent with VOC personnel archival data, the employment of young children (< 10 years) was unlikely^7–9^. The ^87^Sr/^86^Sr and *δ*^13^C isotope data from the permanent first molar of the 3- to 6-year-old child was consistent with local expectations (^87^Sr/^86^Sr = 0.711553; *δ*^13^C_VPDB_ = − 10.6‰; *δ*^18^O_VPDB_ = − 5.4‰); therefore, it is possible that the child had local origins. However, as this combination of isotope data could also be reflective of alternative origins, it is also possible they may have been the child of a passenger transiting through Simon’s Town aboard a VOC, or other company, ship.

In addition to this data, the expectation in a typical cemetery populace would be to find higher inclusions of infant burials considering this period was reported as having a relatively high infant mortality rate, with up to 27% of infants not surviving their first year^12^. While mortality rates can be high in young children, these rates tend to decrease by adolescence (12–20 years old); therefore, we expect a general or residential population to have a higher representation of non-adults, and in particular young children (< 12 years old). In contemporary Cape Town sites, the total non-adult population varies depending on burial site context. For example, non-adult representation at the Cobern Street burial site was approximately 30% (*36*/119), and at the Marina Residence hospital burial site in Cape Town, approximately 8% (*6*/78)^11^. Comparatively, the 3% non-adult representation in the Simon’s Town burial site more closely resembled the Marina Residence demographic. Reviewing available VOC archival data^9^ demonstrated that while non-adult individuals were employed, they were not aboard VOC ships in large numbers. Only 2% of all listed personnel and passengers aboard VOC ships were categorised as “*Jongen*”, which referred to "boys" aged between 10 to 17 years, employed as assistants for various specialist roles on board the ship. Overall, the age and biological sex results from this research represented the expected demographic of a VOC hospital burial site.

The predominant burial style was supine with arms alongside or crossed over the body. Regarding associated material culture, there was evidence in some burials of small fragments of textile, rope, and small pins, suggesting enshrouding prior to interment. Few individuals were found with clothing buttons on or near the torso, implying some individuals were fully clothed at time of burial. Some associated artefacts supported a link between the burial ground and the Dutch colonial era (> 1652 CE). Finds such as European coins (Dutch VOC, French) dating to the seventeenth and eighteenth centuries, a trade bead necklace, and numerous fragmentary pipe stems and bowls (with a Dutch maker’s mark) were found throughout the site.

There were no instances of intentional dental modifications in the form of decorative filing or tooth evulsion as prevalent amongst numerous African^27,28^ and Indian Ocean basin^29^ populations. It must be noted that Cape Town would have already had a high degree of cultural admixture by the 1700s CE^11,49^. Dental modification increased in frequency across South Africa between the eighteenth and nineteenth centuries, particularly in the West, as associated with the increased population of enslaved people from other African and Indian Ocean nations. Therefore, if the buried population included the burials of enslaved people, it was expected to find intentional dental modification in the form of chipping or evulsion as was found at other VOC Cape region sites^10,13,34,49,50^. The only modifications to dentition observed were distinctly notched grooves from pipe smoking in four individuals, occurring from the persistent clenching of clay pipes. Notched grooves were supported by the presence of clay pipe fragments, some directly associated with the burials. As clay pipe use was most prevalent between the sixteenth and nineteenth centuries^51^, this observation was consistent with the period during which the VOC hospital was in use.

The strontium isotope data of all 43 individuals analysed are compatible with known ^87^Sr/^86^Sr ranges for multiple countries of interest, including South African, European, and some young volcanic Indian Ocean basin regions. Of note was that of all 43 ^87^Sr/^86^Sr, no individuals exhibited extremely radiogenic ratios (> 0.7280), as have been identified in other analysed Cape Town sites, which has been suggested to indicate enslaved persons from regions such as Mozambique^10,52^. When considering the multi-isotope data distributions, six individuals (including both potential non-local and local) presented distinctly higher or lower ratios or values from the general sampled individuals and thus merit a more detailed discussion.

Burial 66 exhibited the highest ^87^Sr/^86^Sr of 0.715812 (Fig. 3), a *δ*^18^O_VPDB_ value of − 3.4‰, and a *δ*^13^C value representing a mixed C_3_–C_4_ diet (*δ*^13^C_VPDB_ = − 7.9‰). The ^87^Sr/^86^Sr of burial 48 (0.710458) was within the expected local Cape Town range, with a *δ*^13^C_VPDB_ value of − 10.6‰ indicated a mixed C_3_–C_4_ diet. Yet, burial 48 exhibited the lowest *δ*^18^O_VPDB_ of − 10.0‰. Low *δ*^18^O values may correlate with regions far from the equator, in higher altitudes, further distance from the sea, or areas with heavy precipitation. Therefore, it is possible that burial 48 was non-local considering Cape precipitation data.

Burial 42B had a low ^87^Sr/^86^Sr of 0.707589, but high *δ*^18^O–*δ*^13^C values. The oxygen isotope value for this individual was the highest in the range at *δ*^18^O_VPDB_ = − 3.6‰. Similarly, their *δ*^13^C_VPDB_ value was second highest at − 1.9‰, representing a C_4_ dominant diet. Further, burial 43D had the lowest ^87^Sr/^86^Sr of 0.707409, with a *δ*^18^O_VPDB_ value of − 5.9‰, and a *δ*^13^C_VPDB_ value representative of a mixed C_3_–C_4_ diet (− 9.4‰). Burial 43A also exhibited a lower ^87^Sr/^86^Sr of 0.708839, with a *δ*^13^C_VPDB_ value of − 11.9‰ (mixed C_3_–C_4_ diet) and a *δ*^18^O_VPDB_ value of − 6.7‰. Such low ^87^Sr/^86^Sr are suggestive of regions with a younger geology^10,52,53^ or with increased influences from marine derived strontium, and are not considered characteristic of the Cape Town region.

Three further individuals exhibited high *δ*^13^C values indicative of dominant C_4_ diets. Burial 105’s ^87^Sr/^86^Sr of 0.709102 was within the local range, with a *δ*^18^O_VPDB_ value of − 6.5‰, yet *δ*^13^C_VPDB_ value of − 5.3‰ is suggestive of a mixed diet with significantly higher C_4_ inclusion than is expected for the Cape region. Burial 72 also had an ^87^Sr/^86^Sr within the expected local Cape Town range (0.710528), and a *δ*^18^O_VPDB_ of − 6.6‰, yet also indicates a high *δ*^13^C_VPDB_ value of − 3.8‰, suggesting a diet more dominant in C_4_ plants. Finally, burial 138 exhibited a local ^87^Sr/^86^Sr (0.712174), with a *δ*^18^O_VPDB_ of − 5.0‰. The *δ*^13^CV_PDB_ value of burial 138 was the highest amongst the analysed sample (*δ*^13^C_VPDB_ = − 1.3‰), and like burial 42B, suggests a C_4_ dominant diet.

While exact origins cannot be identified, burials 42B, 72, 105, and 138 originated from regions where diets were dominant in C_4_ plants. The VOC occupation of the Cape region introduced C_4_ plants to the predominantly C_3_ diet. Yet, most research on the area has found that local diets were C_3_ dominant to mixed C_3_–C_4._ A pure C_4_ diet has been considered indicative of migration from elsewhere into the Cape in other contemporary sites^10,34,54^, therefore, we can conclude that these individuals did not originate from the Cape region.

Finally, it is worth briefly highlighting that a clear difference may be observed between the boxplots comparing the ^87^Sr/^86^Sr, *δ*^18^O_VPDB_ and *δ*^13^C_VPDB_ data between Simon’s Town (this research) and the contemporary Cobern Street site. Differences included more confined ^87^Sr/^86^Sr and *δ*^13^C values, and minimal overlap in the *δ*^18^O data. However, the *δ*^18^O was assessed with discretion due to intra-dental variations or variations introduced by pretreatments^43,44^. This is of note as the Simon’s Town samples were pretreated, whereas the Cobern Street samples were not. When comparing the data of all three isotopes between the sites, independent two-tailed *t*-tests highlighted statistical differences between the two populations for ^87^Sr/^86^Sr (Cobern Street: M = 0.71517, SD = 0.00705, this research: M = 0.71024, SD = 0.00146); *t*(40) = 4.241, *p* = < 0.001) and *δ*^13^O (Cobern Street: M = − 3.55, SD = 1.05, this research: M = − 6.31, SD = 1.23; *t*(79) = 10.874, *p* = < 0.001), while the *δ*^13^C values were not statistically different (Cobern Street: M = − 9.36, SD = 3.47, this research: M = − 10.33, SD = 2.76); *t*(71) = 1.369, *p* = 0.175). The higher ^87^Sr/^86^Sr and *δ*^13^C_VPDB_ values from Cobern Street were combined with dental modifications to identify enslaved individuals who were displaced from outside of the Cape region^13,34^. However, considering the sites are contemporary, there are notable and significant population differences between those buried at Simon’s Town *versus* those at Cobern Street. While further comparable data from the region would be required to make stronger inferences between sites, the statistically significant differences in the multi-isotope data between these two contemporary VOC-related sites suggested that the populations were dissimilar in geographical origins.

Taking all available data into account, the overall combined isotope results suggested that the 43 analysed individuals exhibited a combination of possible local individuals, and 16% (*7*/43) of geographically diverse non-local individuals in the Cape Town region. Directly comparable isotope data from a contemporary VOC related burial ground highlighted statistically significant differences between the ^87^Sr/^86^Sr and *δ*^18^O values of Simon’s Town and Cobern Street populations. The few associated artefacts found with burials were congruent with the Dutch colonial era. However, the age and sex demographics more closely resembled the contemporary Marina Residence hospital burial ground in Cape Town. As only males were identified through peptide analysis (*25*/25), this strongly supported an atypical burial population, such as a location catering to VOC personnel.

## Conclusion

This research aimed to obtain as much information as possible about the interred individuals and their buried context using informative methodologies, including macroscopic observations and biochemical techniques. Due to poor preservation of the skeletal remains, it was impossible to conduct standard osteological techniques, apart from larger observations on pathological conditions in a few individuals. Observed conditions included residual rickets and dental conditions such as calculus, caries, enamel hypoplasia, and antemortem tooth loss. Additional non-masticatory wear was observed in the form of anterior and premolar notching from clay pipe use. The site demographic was predominantly adult (> 20 years) with six non-adults. Moreover, 25 individuals assessed for enamel peptide analysis presented with both AMELX and AMELY sequences which determined that all 25 individuals were biologically male. The most prevalent burial style was supine with arms down either side of the body or flexed over the chest or torso. The material finds from the site included fragments of clay pipes, European trade coins, trade beads, buttons, and textile remnants.

As isotope analyses could only be applied to tooth enamel; a multi-isotope approach using strontium (^87^Sr/^86^Sr), as well as oxygen (*δ*^18^O_VPDB_) and carbon (*δ*^13^C_VPDB_) from enamel carbonate was applied. Our findings demonstrated that a combined isotope approach was valuable in identifying a geographically diverse population, with the addition of *δ*^18^O and *δ*^13^C being of use in identifying non-locals. At least 16% of the analysed population (*7*/43) were non-local to the Cape Town region, supporting the link between this site and the former VOC hospital’s burial ground.

The combined methodological approach to this site elucidated many aspects of the possible origins and contexts of the buried individuals. The findings suggested that there remained valid possibilities to glean information when faced with an unknown or limited context, ideally where a sizable sample of a burial site may be assessed. There are few published reports on VOC sites with human remains, as many passengers and ships were lost at sea. As such, this discrepancy between VOC-related deceased individuals and low recovery rates is an example of the need to prioritise the assessment of remains suspected from a fragmentary context such as the Simon's Town site. Moreover, our study demonstrates the first known multimethod approach in bioarchaeological literature that combined historical archives, archaeology, macroscopic osteology, multi-isotope, and peptide analysis to interpret an individualised and overall context for unknown remains. Consequently, our study contributes to VOC, Dutch, and South African history during the period of colonisation, shedding light on VOC hospital burial practices in South Africa.

This study further demonstrated that a combined biochemical and osteological approach for archaeological remains may elucidate origins and burial contexts, chiefly when remains are poorly preserved with no surviving organic material. We stress that assessing more of the sampled individuals may alter current interpretations of the buried individuals, as the individuals analysed in this research only represent a portion of the excavated site. However, at present these data align with the hypothesis of a VOC hospital burial ground, and the lack of results that deviated from this hypothesis further supported these interpretations. Nevertheless, these findings should be reassessed upon publishing additional bioavailable VPDB isotope values and isoscapes for the regions of interest, and we encourage researchers to prioritise studies in this regard to improve the accuracy of global archaeological comparisons on human origins and mobility.

Considering the results of this research in the context of the archival, historical, and archaeological data, we conclude that the multi-method approach found evidence that the skeletons are linked to the VOC period. Specifically, the consistent burial styles, the lack of dental modifications, the exclusively male proteomics results, and the isotope results revealing a mix of non-local and possible local origins, all strongly suggest the site is the burial ground of the former VOC hospital.

## Methods

### Fieldwork and sampling

A Cape Town-based archaeological company, ACO Associates (field director: Tim Hart), completed the excavation of the site. A forthcoming site report detailing the excavation will be publicly available. The skeletal remains were poorly preserved; therefore, osteological observations were limited to dental modifications or gross pathological conditions. Non-adults were aged by assessing dental eruption, formation and epiphyseal fusion^22–25^. Pathological conditions were identified, where possible. Notably, skeletal pathological conditions were only identified if they were primarily apparent on multiple or gross skeletal elements (e.g., residual rickets, trauma) due to poor preservation affecting visibility. Sampling involved the extraction of two teeth per individual, with one tooth specifically for aDNA analysis to avoid contamination, using stainless steel tools. A total of 84 individuals were sampled. Permits were obtained for destructive sampling/analysis, and for the export of human remains outside of South Africa (see “Ethics declaration” section for permit information). The first molars of the permanent dentition were preferentially selected for elucidating local vs. non-local origins through ^87^Sr/^86^Sr, *δ*^18^O, and *δ*^13^C isotope analysis, considering the first molar crown cusps begin to form around birth, with crown completion around 3 years old**.** Where the first molar was absent or damaged, the closest adjacent tooth was sampled, preferentially the second molar with crown completion occurring around 7–8 years old^24,25^. Tooth extraction was performed in situ or in a clean, local museum workplace if individuals had already been exhumed. As samples were expected to be analysed for aDNA, a clean protocol was followed involving personal protective equipment in the form of FFP2 respirator masks, hairnets, and two layers of nitrile gloves, with the outer layer being changed between individuals to avoid potential DNA contamination. Samples were immediately stored in sterilised 15 mL and 50 mL CELLSTAR^®^ polypropylene sample tubes.

### Preliminary tests for collagen and aDNA

Several preliminary tests on dentine and tooth roots were conducted for ^14^C dating, carbon and nitrogen isotopes in collagen, and aDNA, but all tests revealed that neither collagen nor aDNA had preserved.

### Proteomics

Peptide analysis uses tooth enamel to assess the sex-chromosome encoded enamel-forming proteins, amelogenins, which are laid down during the development of our teeth, and therefore inform us of biological sex. Amelogenin proteins are sexually dimorphic markers of X and Y chromosomes and can therefore indicate whether an individual is chromosomally male or female^55^.

A total of 25 samples were processed for targeted proteomic (LC–MS) analysis, where sample protocols required uncleaned enamel chips or complete tooth crowns^55,56^. The enamel surface was abraded to remove surface contaminants and washed in Milli-Q water. Peptide extraction and analysis was performed^55^ with enamel etching in 60 μL of 5% (v/v) HCl extended to 60 min. Peptides were purified using C18 resin-loaded ZipTips with the final 60% acetonitrile/0.1% formic acid elution lyophilised. Each sample was redissolved in 0.1% aqueous TFA (10 µL), centrifuged (100×*g*, 2 min) and 8 µL of the supernatant transferred into a tapered LC vial. Samples were analysed with a Dionex U3000 nanoLC coupled to a ThermoFisher LTQ FT Ultra Mass Spectrometer containing a nano-ESI source. An injection volume of 2 µL was loaded onto a C18 Pepmap300 loading column (10 mm, 300 Å, 5 µm particle size) using 0.1% aqueous TFA as a loading solvent. Sample separation was performed using a C18 Pepmap300 column (150 mm × 75 µm, 300 Å, 5 mm particle size) with a gradient of two mobile phases: mobile phase A (5% acetonitrile, 0.1% formic acid) and mobile phase B (95% acetonitrile, 0.1% formic acid). Peptides were eluted using a 30 min linear gradient of mobile phase B from 0 to 55% at a flow rate of 0.4 µL/min followed an increase to 90% B over 0.1 min, which was held for 5 min before returning to 0% B over 0.1 min for a column equilibration period of 25 min. The nano-ESI source was operated a voltage of 1.7 kV in the positive ion mode. The (entrance) capillary temperature was set at 275 °C, with inner capillary voltage value set on 37 V and tube lens value of 145 V. Three data channels were acquired. Full scan FTMS spectra were acquired over a 400–1200 *m/z* range at a nominal resolution of 100,000 (at *m/z* = 400). MS/MS channels were set to acquire precursor ions at *m/z* 440.2 and *m/z* 540.3, with a window of *m/z* 8, corresponding to the 2+ ions of the AMELY and AMELX peptides, respectively. Precursor ions were fragmented in the ion trap of the spectrometer using He collision gas at a nominal collision energy of 35. Product ions were detected in the ion trap. The instrument was controlled, and data visualised by Xcalibur software (Thermo Fisher). The presence of AMELX and AMELY peptides was determined by plotting chromatograms for the transition *m/z* 540.3 to 714.4 (characteristic for AMELX), and *m/z* 440.2 to 645.4 (characteristic for AMELY).

### ^87^Sr/^86^Sr, *δ*^18^O, and *δ*^13^C isotope analysis

Analysis of strontium (^87^Sr/^86^Sr), oxygen (*δ*^18^O_VPDB_) and carbon (*δ*^13^C_VPDB_) isotopes are established methods in archaeology to investigate geographic origins, mobility, and dietary habits of past organisms^47,57–61^. Oxygen and carbon, classified as light isotopes, are influenced by the diet and fractionation processes occurring within the body. Conversely, strontium isotope ratios which are classified as heavy isotopes, are indicative of the underlying geology without undergoing any fractionation processes within the body^38,62^. Due to the predominant mineral content in human dental enamel, it is particularly beneficial in archaeological analysis due to its resilience to diagenetic changes^63,64^. As the interest of this research focuses local vs. non-local origins, the first (M1) and second (M2) molars were selected for sampling. During amelogenesis of these teeth, the individual's dietary intake containing strontium, oxygen and carbon isotopes representative of their geographical environment and diet, are incorporated into the enamel’s calcium hydroxyapatite (HAP, chemical formula; Ca_10_(PO_4_)_6_(OH)_2_)^64–69^. Strontium can be incorporated into HAP through substitution at the calcium (Ca) sites, while oxygen and carbon can be incorporated via structural carbonate (–CO_3_) substitution at both –PO_4_ and –OH sites^64,70^. As HAP does not remodel, the Sr and carbonate substitution sites in HAP represent the isotopic ratios and values incorporated into the enamel during early childhood (during the first 0–3 and 3–8 years of life for M1 and M2 respectively)^24,63^. Consequently, strontium ratios and oxygen isotopic values from human teeth are representative of the underlying geology and water which are incorporated into the body through diet and imbibed water.

Carbon isotope values can broadly infer diet due to isotopic differences between plants utilising C_3_ (e.g., wheat, most fruits, and vegetables), C_4_ (e.g., millet, sorghum, maize) and CAM (crassulacean acid metabolism, e.g., pineapple, vanilla, and succulents) photosynthetic pathways^71–73^. CAM plants have not been explicitly discussed in this research due to the complexity of their mechanisms, as they can produce similar dietary *δ*^13^C values to both C_3_ and C_4_ pathway plants. The inorganic (apatite) components of human tissue are analysed in this research and represent the total diet, inclusive of the protein component that is typically represented by collagenous dietary isotope analysis^74,75^.

A total of 43 individual samples were taken for ^87^Sr/^86^Sr, *δ*^18^O, and *δ*^13^C isotope analysis following preparation protocols outlined by Kootker et al.^52^. Enamel powder was sampled at the Faculty of Archaeology chemistry laboratory at Leiden University, The Netherlands. The surfaces of all sampled cusps were abraded with a Dremel rotary tool to remove external organics. Then, approximately 10 mg of enamel powder was removed in a band across the height of the crown (from the cementoenamel junction to the occlusal surface) using a Dremel rotary tool with diamond burr accessories. Initial indicators of enamel preservation were considered at various stages of sampling by assessing hardness of tooth enamel, response of enamel to drilling, and colour of raw enamel powder^69^. As all drilled teeth had well-preserved enamel, and the duration of interment was expected to be no more than 300 years, no further preservation checks (i.e., FTIR or FTIR-ATR) were deemed necessary. Each sample was treated with 1 mL of a 3% NaOCl solution at room temperature for 24 h, followed by four Milli-Q^®^ water rinses. Then, 1 mL of acetic acid–Ca acetate buffer (pH = 4.75) solution was added for at room temperature for 24 h, followed by the same four Milli-Q^®^ wash rinsing procedure. The samples were transferred to the department of Earth Sciences at the Vrije Universiteit Amsterdam, The Netherlands, where approximately 2 ± 1 mg of enamel powder was subsampled for ^87^Sr/^86^Sr and transferred to the USA class 100 clean laboratory at the Department of Earth Sciences for Sr purification. For combined *δ*^18^O and *δ*^13^C, 0.3 mg ± 10% was subsampled in clean Exetainer^®^ vials and transferred to the stable isotope facility. Strontium column extraction and sample loading were performed following previously published protocols^52^.

The Sr isotope compositions were measured using a Thermo Scientific™ Triton Plus™ thermal ionisation mass spectrometer (TIMS) housed at the Vrije Universiteit Amsterdam. The strontium ratios were determined using a static routine and were corrected for mass fractionation to an ^86^Sr/^88^Sr of 0.1194^76^. The NIST^®^ SRM^®^ 987 standard averaged 0.710258 ± 0.000008 throughout this study (*n* = 36, 2 s). The intermediate precision over the period 2017–2021 using the same method was 0.710254 ± 0.000018 (*n* = 433, 2 s). All measurements were normalised to an accepted value of 0.710240. The total procedural blanks (*n* = 3) contained less than 56 pg strontium. The ^87^Sr/^86^Sr are reported plus ± 2 standard error (2SE), representing the analytical uncertainty calculated from 240 cycles of 8.1 s integration time (12 blocks of 20 cycles) within each run.

For *δ*^18^O and *δ*^13^C, the enamel samples were flushed with helium gas, then reacted with orthophosphoric acid (H3PO4) (100%) at 45 °C for 24 h. The isolated carbon dioxide (CO2) was analysed using a Thermo Finnigan GasBench II preparation device interfaced with a Thermo Finnigan Delta + mass spectrometer at the Earth Sciences Stable Isotope laboratory, Vrije Universiteit Amsterdam, The Netherlands. The data were normalised to the Vienna Peedee Belemnite (VPDB) scale using an in-house carbonate reference material (VICS) calibrated against NBS19 and LSVEC-certified reference materials. The international control standard IAEA-CO1 was used to check instrument performance (mean − 2.36‰, *n* = 8 for the Simon’s Town data (2022), mean − 2.46‰, *n* = 14 for the Cobern Street data (2013)). The reproducibility of IAEA-CO1 during the analytical session was ± 0.12‰ (1σ) for both sessions. Both *δ*^18^O and *δ*^13^C are expressed relative to Vienna Pee Dee Belemnite (VPDB) standard in per mil.

### Ethics declarations

Permits were obtained for the destructive sampling, analysis, and export of human skeletal remains outside of South Africa. This was approved by the South African Heritage Resources Agency (destructive sampling and analysis under Section 35(4) of the National Heritage Resources act, 1999 (Act 25 of 1999) and Regulation 3(3) of PN 298 (August 29, 2003) under CASE# 18061907SB0704E, and export permit under Section 32(19) of the National Heritage Resources Act (Act 25 of 1999), under CaseID: 14380), with the permit holder being Tim Hart.

### Supplementary Information

Supplementary Table S1.

## Data Availability

All data are available upon request from the corresponding author (j.olszewski@hku.hk), Rachael A. Hall (r.a.hall@arch.leidenuniv.nl) or Martijn Manders (m.manders@cultureelerfgoed.nl). The relevant data are also securely stored in the online Dutch research repository DANS, as well as the public database Maritime Stepping Stones; https://mass.cultureelerfgoed.nl/. Forthcoming excavation reports from this site will be publicly available through the South African Heritage Resources Agency SAHRIS online database; https://sahris.sahra.org.za/. The isotope data that support the findings of this study are openly available in IsoArcH^77^. Further data collection of additional elements of the skeletal remains is not possible, as the excavated human remains have been reburied in the Old Burying Ground, Seaforth, in Simon’s Town, South Africa.
