# Supplementary material for: Osteological, multi-isotope and proteomic analysis of poorly-preserved human remains from a Dutch East India Company burial ground in South Africa
Source: Sci Rep. 2023 Sep 6;13:14666. doi: 10.1038/s41598-023-41503-9 (PMC10482838; doi:10.1038/s41598-023-41503-9)
Supplement: Supplementary file 1 — Supplementary Table S1. [file 41598_2023_41503_MOESM1_ESM.pdf]

Osteological, multi-isotope and proteomic analysis of poorly-preserved human remains from a Dutch East India Company burial ground in South Africa

Judyta Olszewski*, Rachael A. Hall, Lisette M. Kootker, Neil Oldham, Robert Layfield, Barry Shaw, Leon Derksen, Martijn Manders, Tim Hart, Sarah A. Schrader

*Corresponding author: j.olszewski@hku.hk

Supplementary Table S1: Osteological and isotope data of one deciduous tooth and first molars from 35 individuals from the Cobern Street, South Africa burial site. Osteological, Sr, and C data from prior publications (see references), and O-C data presented as part of this study.

Burial #	UCT #	Age (years)	Sex*	Element #	$^{87}Sr/^{86}Sr$	2SE	$\delta^{13}C$ / ‰ (apatite, VPDB)	s	$\delta^{18}O$ ‰ (apatite, VPDB)	s	Reference
3	460	20	M	16	0.71375	0.00001	−9.7	0.1	−2.6	0.1	Sr - Kootker et al. 2016; O-C - this study
4	458.1	17 - 18	F	16	0.71581	0.00001	−13.0	0.1	−4.4	0.1	Sr - Kootker et al. 2016; O-C - this study
10	498	35 - 40	F	26	0.71195	0.00001	−11.1	0.1	−3.9	0.1	Sr - Kootker et al. 2016; O-C - this study
12	500	35 - 40	M	46	0.70862	0.00001	−11.5	0.1	−6.3	0.1	Sr - Kootker et al. 2016; O-C - this study
13	501	30 - 40	M	46	0.71350	0.00001	−9.9	0.1	−2.5	0.1	Sr - Kootker et al. 2016; O-C - this study
14	502	45 - 55	F	46	0.71274	0.00001	−12.8	0.2	−4.1	0.2	Sr - Kootker et al. 2016; O-C - this study
15	504	25	M	46	0.71328	0.00001	−10.4	0.1	−4.0	0.1	Sr - Kootker et al. 2016; O-C - this study
18	508	> 50	F	26	0.71465	0.00001	−5.5	0.1	−3.3	0.1	Sr - Kootker et al. 2016; O-C - this study
20A	510+	25 - 30	M	16	0.72738	0.00001	−5.6	0.1	−4.2	0.1	Sr - Kootker et al. 2016; O-C - this study
20B	511+	16	F	46	0.71675	0.00001	−2.6	0.1	−3.8	0.2	Sr - Kootker et al. 2016; O-C - this study
20C	512	1.5 - 2	-	71/2 *or* 81/2	0.71194	0.00001	−8.7	0.1	−2.5	0.1	Sr - Kootker et al. 2016; O-C - this study
21	514	25 - 35	F	46	0.71219	0.00001	−6.8	0.0	−2.9	0.1	Sr - Kootker et al. 2016; O-C - this study
23	516	17 - 19	F	36	0.71211	0.00001	−3.4	0.1	−2.6	0.1	Sr - Kootker et al. 2016; O-C - this study
27B	521	40 - 55	M	26	0.71900	0.00001	−11.8	0.1	−2.6	0.2	Sr - Kootker et al. 2016; O-C - this study
28	522	50	F	46	0.71267	0.00001	−4.2	0.1	−1.9	0.1	Sr - Kootker et al. 2016; O-C - this study
34	528	14-15	F	46	0.71555	0.00001	−13.4	0.1	−3.4	0.1	Sr - Kootker et al. 2016; C - Mbeki et al. 2021; O - this study
40	535+	12	F	46	0.72803	0.00001	−3.6	0.1	−3.6	0.1	Sr - Kootker et al. 2016; O-C - this study
41	536	35 - 50	M	36	0.71822	0.00001	−11.9	0.1	−3.2	0.1	Sr - Kootker et al. 2016; O-C - this study
44	542	40 - 50	F	46	0.73407	0.00001	−11.5	0.1	−3.9	0.2	Sr - Kootker et al. 2016; O-C - this study
46	544	35 - 50	F	16	0.72015	0.00001	−12.9	0.1	−4.0	0.1	Sr - Kootker et al. 2016; O-C - this study
47	545	30 - 40	M	46	0.71260	0.00001	−10.1	0.1	−4.0	0.1	Sr - Kootker et al. 2016; C - Mbeki et al. 2021; O - this study
49	547	30 - 35	M	26	0.72830	0.00001	−6.7	0.1	−5.5	0.1	Sr - Kootker et al. 2016; O-C - this study
50	548+	35 - 50	M	46	0.70639	0.00001	−4.0	0.1	−3.1	0.1	Sr - Kootker et al. 2016; O-C - this study
51	549	35 - 40	M	26	0.71017	0.00001	−12.7	0.1	−3.2	0.2	Sr - Kootker et al. 2016; O-C - this study
52	550+	25 - 35	F	46	0.70921	0.00001	−4.6	0.2	−3.9	0.2	Sr - Kootker et al. 2016; O-C - this study
54	552	30 - 35	M	46	0.71027	0.00001	−12.1	0.1	−3.5	0.2	Sr - Kootker et al. 2016; O-C - this study
56	554	35	M	46	0.71387	0.00001	−11.9	0.2	−2.3	0.2	Sr - Kootker et al. 2016; O-C - this study
58	556	35 - 40	F	32	0.71233	0.00001	−6.6	0.1	−2.6	0.1	Sr - Kootker et al. 2016; O-C - this study
60	558+	30	F	36	0.73605	0.00001	−2.2	0.1	−4.0	0.1	Sr - Kootker et al. 2016; O-C - this study
61	559	35	M	46	0.71183	0.00001	−11.0	0.2	−3.6	0.2	Sr - Kootker et al. 2016; C - Mbeki et al. 2021; O - this study
32	526	50 - 60	M	46	0.71225	0.00001	−11.3	0.1	−5.9	0.2	Sr - Kootker et al. 2016; O-C - this study
57	555	20 - 30	F	36	0.71011	0.00001	−13.1	0.1	−2.0	0.1	Sr - Kootker et al. 2016; O-C - this study
59	557	40	M	36	0.70600	0.00001	−10.2	0.1	−4.8	0.1	Sr - Kootker et al. 2016; O-C - this study
65	563	22 - 25	F	26	0.70921	0.00001	−11.5	0.1	−5.0	0.1	Sr - Kootker et al. 2016; O-C - this study

2SE = 2 standard error, s = standard deviation

* Osteological sex, see references.

Kootker, L. M., Mbeki, L., Morris, A. G., Kars, H. & Davies, G. R. Dynamics of Indian Ocean Slavery Revealed through Isotopic Data from the Colonial Era Cobern Street Burial Site, Cape Town, South Africa (1750-1827). PLOS ONE 11, e0157750 (2016).

Mbeki, L., Kootker, L. M., Laffoon, J. E., Davies, G. R. & Kars, H. A dietary assessment of colonial Cape Town's enslaved population. Archaeological and Anthropological Sciences 13, 17 (2021).
